# Direct Laser Patterning of CdTe QDs and Their Optical Properties Control through Laser Parameters

**DOI:** 10.3390/nano12091551

**Published:** 2022-05-04

**Authors:** Francesco Antolini, Francesca Limosani, Rocco Carcione

**Affiliations:** 1 Fusion and Technologies for Nuclear Safety and Security Department, Physical Technologies for Safety and Health Division, Photonics Micro and Nanostructures Laboratory, ENEA C.R. Frascati, via Enrico Fermi 45, 00044 Frascati (RM), Italy; 2 Department of Information Engineering, Polytechnic University of Marche, Via Brecce Bianche, 1, 60131 Ancona, Italy; f.limosani@univpm.it; 3 INFN-National Laboratories of Frascati, Via Enrico Fermi, 54, 00044 Frascati, Italy; 4 Consiglio Nazionale delle Ricerche, Institute of Materials for Electronics and Magnetism (CNR-IMEM), Parco Area delle Scienze 37A, 43124 Parma, Italy; rocco.carcione@imem.cnr.it

**Keywords:** semiconductor quantum dots, CdTe, direct laser patterning, polymer, nanocomposite, fluorescence microscopy, fluorescence spectroscopy

## Abstract

Direct laser patterning is a potential and powerful technique to localize nanomaterials within a host matrix. The main goal of this study is to demonstrate that by tuning some parameters of a laser source, like power and laser pulse frequency, it is possible to modify and tune the optical properties of the generated quantum dots (QDs) within a host matrix of a specific chemical composition. The study is realized by using cadmium telluride (CdTe) QD precursors, embedded in polymethylmethacrylate (PMMA) host matrix, as starting materials. The patterning of the CdTe QDs is carried out by using a UV nanosecond laser source at 355. Fluorescence microscopy and photoluminescence spectroscopy, associated with transmission electron microscopy, indicate that it is possible to obtain desired patterns of QDs emitting from green to red of the visible spectrum, due to the formed CdTe QDs. Preliminary highlights of the CdTe QDs’ formation mechanism are given in terms of laser power and laser pulse frequency (repetition rate).

## 1. Introduction

The localized formation of nanomaterials is a crucial step for the fabrication of devices, like displays with enhanced performances, and also offers new opportunities for new device manufacturing. The most common patterning techniques are photolithography [1], contact [2] and ink-jet printing [3]. Each one of these techniques has its advantages and disadvantages of resolution, costs or throughput. For example, photolithography [4,5] is a well-known method with high throughput; however, it needs several working steps, masks and sometimes the chemistry used during the process can damage the deposited nanomaterials [6]. The contact printing technique, on the other hand, shows good resolution, but it is difficult to exploit for large area deposition, due to limited repeatability [7]. Ink-jet printing is widely used because it is mask-free and is flexible for the deposition of large areas; however, it suffers from limited resolution [3], and the ink and the nozzle for printing need to be carefully selected to prevent frequent clogging. Although these patterning technologies are still under development, other strategies are also explored, and direct laser writing (DLW) is a possible option because it is fast, scalable, and allows both materials synthesis [8] and structuring [9].

The DLW of nanomaterials is defined here as the ability to write arbitrary and programmed paths (patterns), locally formed by nanostructures, in a single step process that involves the use of a laser source [10]. In particular, in this work, the DLW refers to the use of laser light that induces chemical reactions aimed at producing QDs starting from specific substances called precursors dispersed within a suitable host matrix.

In this specific case, a further advantage of the laser technology is related to the inherent nature of the material that can be formed by the laser, i.e., the QDs. The semiconductor QDs [11], indeed, have very promising optical properties concerning their organic and phosphor counterparts. First of all, their sharp emission (FWHM 20–30 nm) make it possible to obtain high color purity [11]. Moreover, the tunability of color emission, without changing chemical composition [12], allows wide representation of the color gamut [13]. The tuning of the optical properties as a function of the QDs’ size, the quantum size effect [14,15], coupled with a relatively simple chemical synthesis is another advantage of QDs: this means that the modification of the optical properties does not require complete changing of the reaction strategy (reagents, solvents, and so on), but it is sufficient just to modify the annealing conditions (time or temperature, for example) [16,17,18]. This property is absolutely important from the technological point of view because time and temperature are control parameters that can be modulated easier than change of path of a chemical reaction, and this advantage can be successfully exploited with laser technology.

In this direction, the combination of DLW technology with the growth of QDs can potentially be exploited to synthesize patterns of nanomaterials with modulable optical properties as a function of the laser writing process (laser power, repetition rate, scan speed, etc.). This type of synthesis has been studied over the years, in particular using a small group of molecules, such as thiolates [19], selenolates [20], and dithiobiureto complexes [21] of cadmium as precursors. In these cases, a UV laser source is used with a wavelength of 266 [22] or 355 nm [23] that is absorbed by the cadmium complex. In such a way, the beam energy is enough to decompose the precursor and form the QDs [24,25].

In all these cases, however, the PL emission of the semiconductor QDs is quite broad ranging from 400 nm to 700 nm. For a real application it would be better to maintain the optical properties of the QDs, tunable within all the visible spectrum, with relatively sharp emission. 

This aim can be achieved by selecting a precursor able to produce CdTe QDs characterized by a bandgap suitable for absorbing and emitting at visible-near infrared (NIR) wavelengths, by modulating the nanometric size of the particles within a small range (2–5 nm) [26,27].

The formation of the CdTe nanocrystals starting from precursors, incorporated within polymer matrices, can occur through a decomposition process of the precursors themselves, induced by heat [16]. In general, the variation of the temperature and the reaction time induces the formation of QDs having different emission colors depending on the size of the nanocrystals (i.e., quantum confinement effect). However, in the processes induced by laser radiation, there is a stringent condition for the synthesis of QDs, because when the precursors do not absorb at the wavelength of the laser source, the chemical reaction triggering the growth of QDs does not take place. To overcome the problem of the transparency of the precursors to the laser light, an alternative way is to introduce a molecule, called a sensitizer, which is capable of absorbing the light emitted by the laser source and transforming it into heat [28,29], which, in turn, induces the chemical reaction of formation of QDs.

The goal of this study is twofold: (i) the extension of the process of laser patterning to precursors that do not absorb laser radiation and (ii) the demonstration of QDs’ formation with tuneable photoluminescence as a function of laser parameters.

The strategy identified in this work to grow the QDs by thermal treatment with laser is to add a sensitizer to the QD precursors in the film that converts the energy of the laser beam in the form of heat.

The second goal, i.e., the formation of QDs emitting from green to red of the visible spectrum, is pursued by using CdTe QDs, because they have a narrow band-gap of 1.5 eV [30] that allows PL emission ranging from green to red within a small variation of particles’ size.

The CdTe QD precursors are formed starting from the cadmium isostearate (Cd(ISA)_2_) and trioctylphosphine-telluride (TOP-Te) included within the polymeric matrix (polymethylmethacrylate—PMMA). These precursors are successfully utilized to study the formation of the CdTe QDs within the PMMA induced by the temperature [16]. So, their combination with a sensitizer, like 2-(2H-Benzotriazol-2-yl)-4,6-ditertpentylphenol) (BZT) [31], that absorbs UV light of the laser and converts it into heat, should induce the formation of the QDs by a thermal process. The process of the QDs’ formation is monitored with a fluorescence microscope, spectrofluorimeter and transmission electron microscopy.

## 2. Materials and Methods

### 2.1. Chemicals and Film Preparations

BZT (2-(2H-Benzotriazol-2-yl)-4,6-ditertpentylphenol), Cadmium oxide (CdO, 99.5%), tellurium (Te, 99.8%), trioctylphosphine (TOP), poly (methyl methacrylate) (PMMA) Mw 120.000, isostearic acid (ISA, technical grade, 90%), chloroform (≥99.8%) were purchased from Sigma-Aldrich (Milano, Italy) and used without further purification.

The synthesis of Cd(ISA)_2_ was carried out according to the procedure reported in the literature, with minor modifications for cadmium carboxylate compounds [32].

The hybrid polymeric films containing cadmium and tellurium precursors were prepared by dissolving PMMA into chloroform to achieve the desired final concentration (100 mg/mL, 200 mg/mL, or 300 mg/mL). A typical protocol for the film formation was arranged by preparing a volume of 1 mL of the polymeric solution where 40 mg, 0.059 mmol, (or 80 mg/mL or 120 mg/mL) of Cd(ISA)_2_ and 2% (*w*/*w* with respect to the polymer) of BZT were added in a 4 mL vial. The mixture was left under stirring for 1 h, at T = 30–35 °C until Cd(ISA)_2_ until it was completely dissolved. Within the Glove Box (ITECO G50), a volume of 16.5 μL (0.0148 mmol) of trioctylphosphine telluride (TOP-Te) solution [33] 0.9 M was added to the PMMA/Cd(ISA)_2_ solution, respecting the molar ratio of Cd and Te to 4:1 [34]. The mixture was taken out of the Glove Box and left stirring again for 10–20 min at T = 30–35 °C to achieve a completely homogeneous solution. 

This solution was used to deposit films by the spin coating technique on glass (25 × 10 mm^2^) for laser treatment or silica substrates (20 × 10 mm^2^) for optical characterization, when necessary. The depositions were performed by using a PoloSpin 150i/200i spin coater. The films were deposited on substrates by dropping 100 μL of the solution over a substrate that was then spin-coated for 45 s at 1000 rpm.

### 2.2. Laser Treatment

The laser treatment was carried out with a Mosquitoo UV diode-pumped solid-state laser at 355 nm (Innolas) with a pulse duration of 10 ns, 1 Watt of maximum power, and pulse frequency (repetition rate) range between 20 kHz and 100 kHz, driven by a computer that allowed regulation of (i) the laser beam movement (speed and position in xy driven by special mirrors), (ii) the laser nominal power (from 20% to 100% of nominal power of 1 W) (iii) the pulse frequency from 20 to 100 kHz and (iv) the number of passes over the same position, called loop counts (LCs). The laser beam at its focus has a diameter of 30 μm.

**Figure 1:**
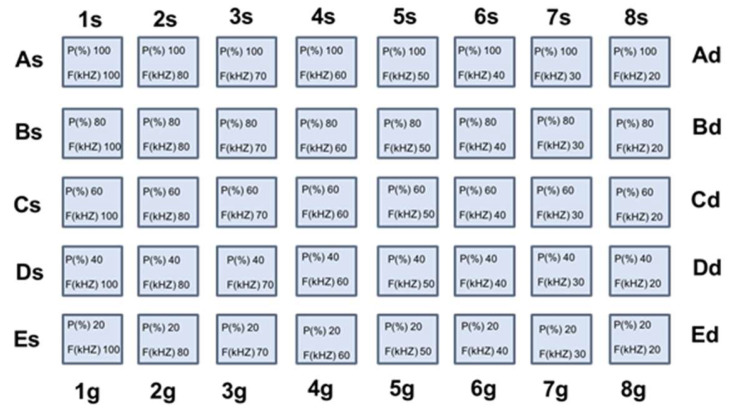
General matrix of laser treatment where two of the main laser parameters, as programmed by computer, are reported: maximum power percentage and pulse frequency.

In a typical direct writing laser experiment a matrix of squares, five rows (A–E), and height columns (1–8) were programmed with a side length of 1 mm. Each square is formed by lines separated by 0.1 mm. The general matrix shown in Figure 1, was used to explore the wider range of power and frequencies i.e., from 100% to 20% of the nominal laser power (1 W) and from 20 kHz to 100 kHz.

The speed of the beam during the writing was set at 20 mm/s or 100 mm/s as specified with the paper, like the number of the loop counts.

The laser power was measured with a power meter for each couple of nominal power and pulse frequency programmed in the computer. For example, the laser power of the square A1 in Figure 1 was obtained by setting the laser nominal power at 100% and laser pulse frequency at 100 kHz. In these conditions, the power meter measured a constant power under the beam, corresponding to 225 mW. This value was used to calculate the fluence and the dose of the A1 square, as reported in the formulae below.

$$\frac{P_{m}\left(\mathrm{mW}\right)}{Freq\;\left(\mathrm{Hz}\right)*Area\;\left({\mathrm{cm}}^{2}\right)}=Fluence\;\left(\frac{\mathrm{mJ}}{{\mathrm{cm}}^{2}}\right)$$

$$\frac{Fluence\;\left(\frac{\mathrm{mJ}}{{\mathrm{cm}}^{2}}\right)*d\left(\mathrm{mm}\right)*Freq\;\left(\mathrm{Hz}\right)}{s\;\left(\frac{\mathrm{mm}}{s}\right)}*LC=Dose\;\left(\frac{\mathrm{mJ}}{{\mathrm{cm}}^{2}}\right)$$

where “*P_m_*” is the measured power in mW of the laser beam at a determined pulse frequency “*Freq*”, measured in Hz, and the “Area” is the area of the beam spot in cm^2^ (7.065 10^−6^ cm^2^ for a spot diameter of 30 μm). The dose was calculated, including the laser spot diameter “*d*” in mm and the beam speed “*s*”. over the film in mm/s and the number of loop counts “*LC*”.

**Figure 2:**
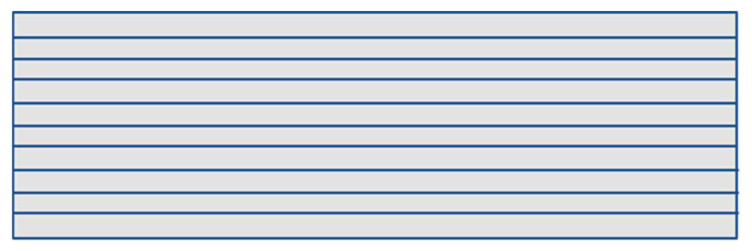
Image of the large area samples obtained drawing lines with distance of 0.1 mm. The size of the large area sample was within a few cm^2^, i.e., 25 mm × 10 mm.

Large area samples were obtained by treating them with specific laser parameters over all areas of the sample. The strategy was to draw lines close to one another until all the film was covered. In general, the distance between the laser lines was set to 0.1 mm (Figure 2).

### 2.3. Fluorescence Microscopy

The samples were deposited on a glass slide, then treated with the laser and finally observed with the fluorescent microscope.

The fluorescence images were obtained with an optical microscope Leica DM 2700M equipped with a color camera DMC2900, and the objective lenses NPLAN EPI 10x/0.25a. The observation of the samples was carried out under white light with a LED source for the Bright Field images and UV excitation of a filtered HB0 100 W lamp. The filter cube (Leica filter cube A) had a UV excitation filter at 340–380 nm, a dichroic mirror at 400 nm, and a long pass filter at 425 nm.

The software Leica LAS X Professional (Leica Microsystems, Wetzlar, Germany) was utilized for image acquisition and elaboration.

### 2.4. Optical Characterization

The optical properties of the film deposited on a silica slide were investigated by UV-Vis absorption and PL spectral measurements.

Absorption spectra were recorded on a Jasco V750 spectrophotometer, in the spectral range of 200–800 nm, an integration time of 0.6 s and slit widths of 1.5 nm.

The PL emission spectra were obtained by using a Fluoromax 4 Plus (Horiba Italia s.r.l., Roma, Italy) spectrofluorimeter equipped with Origin program for data acquisition and analysis in the spectral range from 450 to 690 nm. The excitation wavelength of 350 nm was used with a spectral bandwidth of 1.5 nm for both the excitations and emission monochromators and output cut-off filter at 399 nm to characterize all the samples. The curves were automatically corrected for the spectral response of the detector. 

### 2.5. Transmission Electron Microscopy (TEM)

The morphological and structural properties of the samples were investigated by TEM and High Angle Annular Dark Field- Scanning Transmission Electron Microscopy (HAADF)-STEM analyses. The TEM analyses were performed with a FEI TECNAI F20 microscope (Thermo Fisher Scientific, Milano) operating at 200 keV. The instrument was also equipped with a dispersion micro-analysis of energy (EDS) and STEM accessory. The TEM images were taken in the phase contrast mode (HREM).

The STEM pictures were recorded using HAADF detectors: in this imaging mode the intensity I was proportional to Z^1.7^t, where Z is the mean atomic number and t is the thickness of the specimen.

The samples for TEM observations consisted of QD solution dropped over the TEM grid. The QD solutions were prepared by dissolving the laser-treated film deposited on a glass substrate (25 mm × 10 mm) with 240 μL of chloroform.

## 3. Results

The study of QD formation by laser started with previous research on CdTe formation by thermal treatment within a polymer like PMMA [16]. This study demonstrated that Cd(ISA)_2_ and TOP-Te precursors are suitable to produce CdTe QDs with a size change associated with color emissions from green to red. In the present study, the type of polymer and precursors were the same, except that CdTe QD formation was obtained by laser.

**Figure 3:**
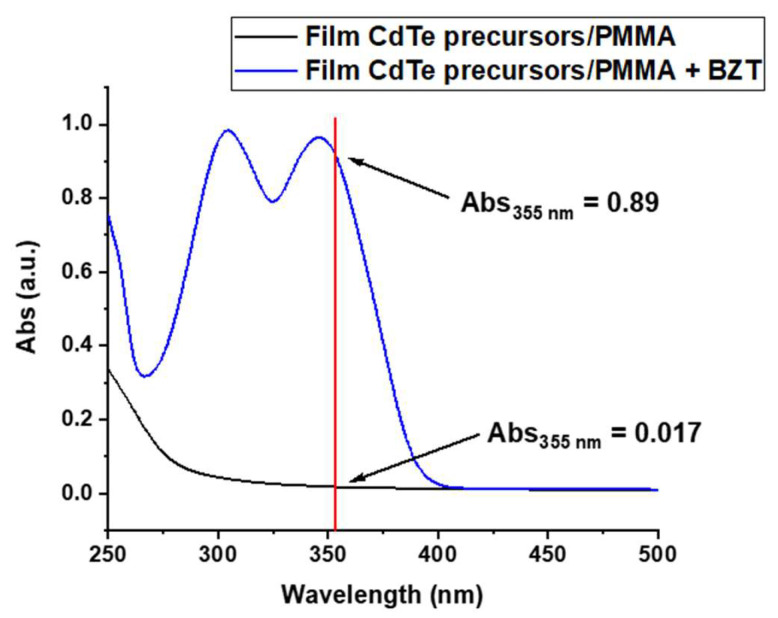
Absorption spectra of CdTe precursor film without BZT (black line) and with BZT (blue line. The different absorption at 355 nm is highlighted to emphasize the role of the BZT within the film.

As the first step of CdTe QD formation by laser, a film of CdTe precursor was structured with the 355 nm nanosecond laser. However, this treatment did not cause any change in the optical properties of the film, because it did not absorb enough laser radiation at 355 nm. To overcome this problem, UV absorber BZT [31] was added to the film so that the laser light energy could be more efficiently absorbed and converted into thermal energy, inducing QDs’ formation. Figure 3 shows the absorption spectra of the CdTe precursor film with, and without, BZT, confirming that the UV sensitizer enhanced, by about 50 times, the absorption of the film at 355 nm.

With this new formulation, i.e., CdTe precursors, BZT and PMMA, it was possible to obtain colored emitting areas by DLW, as shown below. If the film was loaded with BZT, but without CdTe precursors, any colored areas were formed (Figure S1 supporting information). 

Given such considerations, the first issue was to find a range of precursors and polymer concentrations suitable to produce CdTe QDs with color emission. 

The laser parameters, i.e., the nominal power and pulse frequency, were modulated in the widest possible range compatible with the possibilities of the instrument (general matrix search in Table 1), with loop count equal to 3 and beam speed set at 100 mm/s.

**Figure 4:**
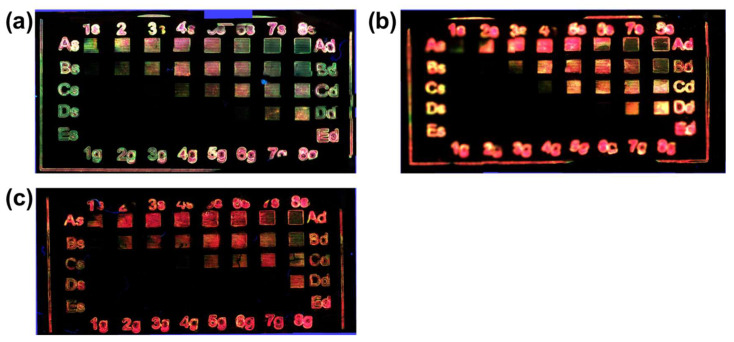
Photo of the laser treated film under epi-fluorescent microscope (excitation with UV filter) with different Cd(ISA)_2_ concentrations: (**a**) 40 mg/mL, (**b**) 80 mg/mL and (**c**) 120 mg/mL. The PMMA concentration is fixed at 300 mg/mL. The side square is 1 mm.

Maintaining constant polymer concentration at 300 mg/mL, the Cd(ISA)_2_ amount was tested at three different concentrations; namely 40, 80 and, 120 mg/mL. The effect of the general matrix laser treatment of these samples is reported in Figure 4a–c, while the laser parameters and the doses involved in the laser structuring are shown in Table 1. The first evidence is that it is possible to get green and red areas just by changing the laser parameters. The second highlight is that when the concentration of the precursor is higher than 40 mg/mL the red color is dominant (Figure 4b,c).

The second set of experiments was carried out by keeping the precursor concentration at 40 mg/mL constant, and changing the polymer concentration from 100 mg/mL to 200 mg/mL and then to 300 mg/mL.

**Figure 5:**
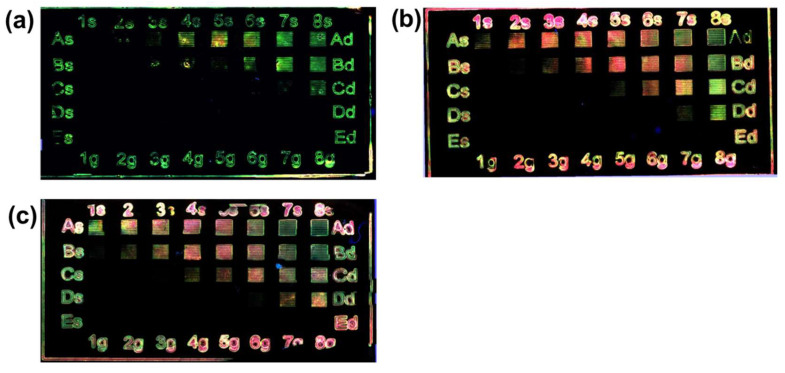
Photo of the Laser treated film under epi-fluorescent microscope (excitation with UV filter) at different PMMA concentration: (**a**) 100 mg/mL, (**b**) 200 mg/mL and (**c**) 300 mg/mL. The Cd(ISA)_2_ is 40 mg/mL. The laser parameters and the doses used are reported in Table 1.

Three films with different polymer concentrations were tested again by using the laser parameters shown in Table 1 (loop count 3 and speed 100 mm/s) and the result is shown in Figure 5. In these images, it is possible to observe that with PMMA 100 mg/mL (Figure 4a) the emission is practically always green, while the green and red squares at 300 mg/mL (Figure 5c) do not have the same quality as at 200 mg/mL (Figure 5b).

From this first set of experiments, it was possible to select the chemical composition suitable to produce color emissions from green to red with acceptable quality, formed by Cd(ISA)_2_ 40 mg/mL and PMMA 200 mg/mL.

In the meantime, Table 1 allowed selection of the ranges of doses (laser parameters) to obtain these color emissions: the red squares in Figure 4b A2–6, B4–6, C6–7 indicate that the laser dose values ranged from 35 J/cm^2^ to 85 J/cm^2^ (pulse frequency between 40–80 kHz in Table 1), while the dose range suitable for green squares is smaller and falls within the doses’ interval of 30–130 J/cm^2^ (pulse frequency 20 kHz in Table 1 and column 8 from A to D of Figure 5b). 

Here it is important to highlight that the green squares were obtained at a pulse frequency of 20 kHz, while the red squares needed higher pulse frequencies (40–80 kHz) to be formed.

A refined search was carried out to improve the quality of red and green light-emitting squares in terms of color brightness and homogeneity of the lines investigated, andalso at higher magnifications.

The refinement of the green emission was done by maintaining the pulse frequency at 20 kHz as a constant, as deduced from Figure 5b, and varying the laser doses from 10 J/cm^2^ to 200 J/cm^2^ by changing the number of the loop count (from 1 to 5), as shown in Table 2.

**Figure 6:**
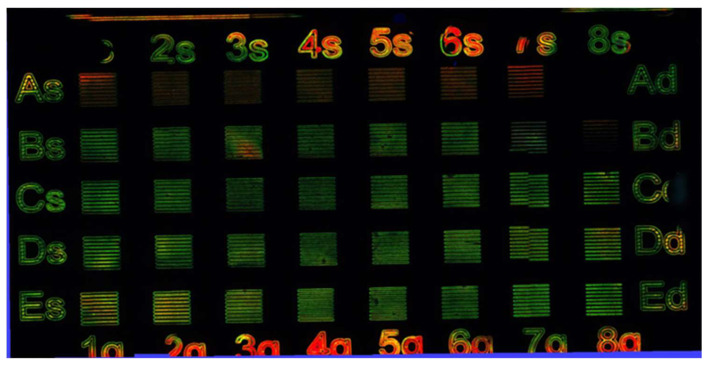
Green search matrix results. The pulse frequency is 20 kHz for all the tested squares with side of 1 mm. The laser parameters and the dose are reported in Table 2.

The results of this treatment are shown in Figure 6. The first row, LC equal to 1, shows that the laser treatment had no effect, or produced very faint red lines (squares A6–7). Only when the laser over-wrote the squares (loop counts from 2 to 5) did the green color begin to appear. From this image, it is possible to observe that the best green squares are the ones produced at lower doses 30–65 J/cm^2^ (laser power between 250–380 mW) with loop counts 3–4 (C6–8 and D6–8). Indeed, at higher doses (D1–3, E1–3) the center of the lines within the square is black (probably the color emitter was destroyed) because the energy transferred to the film was too high (see supporting information Figures S2 and S3).

The refinement of the red areas was carried out with a specific matrix with loop count 1, doses between 7 and 13 J/cm^2^, and pulse frequency between 80 and 100 kHz to avoid any chance of overlapping the green range of doses (30–65 J/cm^2^ Freq. 20 kHz), as shown in Table 3.

**Figure 7:**
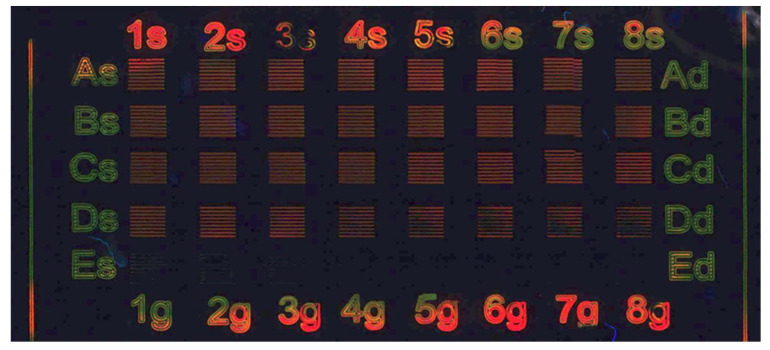
In the red search matrix, the pulse frequency changes from 100 kHz step by step from A1 to C4 at constant power of 100%, while from C5 to E8 the pulse frequency is constant at 100 kHz and the power shift from 100% to 80 % step by step. All the tested squares had sides of 1 mm. The couple dose/pulse frequency is reported in Table 3.

Figure 7 shows that the red color can be achieved at fluences within the range 7.5–13.0 J/cm^2^ with pulse frequency between 80 and 100 kHz and loop count 1 with a speed of 100 mm/s (Table 3).

However, a deeper analysis of the lines forming the squares shown in Figure 7 illustrates that they contain some green emissions (see Figure S4 supporting information). So, maintaining the pulse frequency at 100 kHz and the nominal power at 100% (225 mW) the dose was increased again from 10 to 100 J/cm^2^ by decreasing the speed of the beam from 100 mm/s to 10 mm/s (Table 4). In these conditions, it was possible to obtain good red lines at beam speeds below 30 mm/s (see Figure S5 supporting information), which means a dose between 30 and 100 J/cm^2^ (Table 4).

Finally, to obtain the red pattern a speed of 20 mm/s was selected and the pulse frequency of 100 kHz was kept constant, while doses were changed between 30 to 50 J/cm^2^ (laser power between 150 and 225 mW).

Table 5 resumes all the main parameters to obtain the red and green patterns with good quality.

To carefully check the optical characteristics of the red and green areas and their formations, further studies of the large area samples were carried out. Large area samples are necessary to measure the absorption and PL spectra of the emitting patterns and acquire the TEM images to verify the presence of quantum dots. 

**Figure 8:**
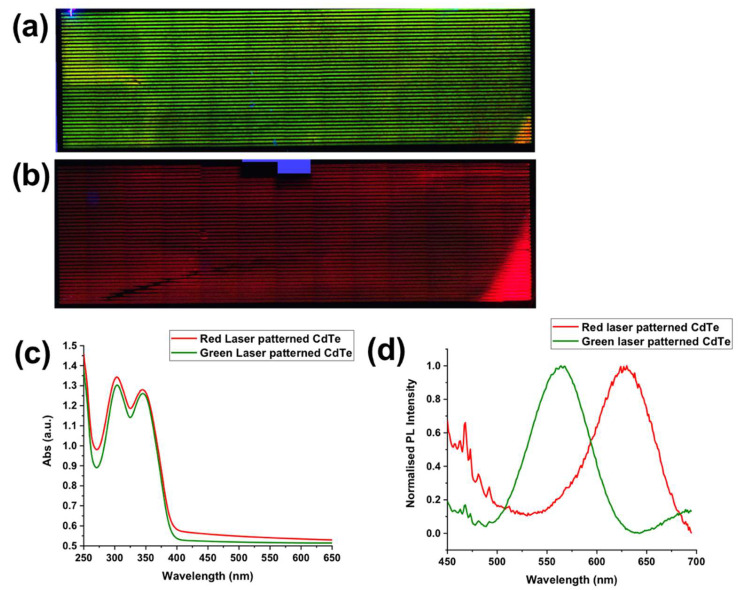
Large area image of the green (**a**) and red (**b**) sample (PMMA 200 mg/mL and Cd(ISA)_2_ 40 mg/mL) acquired with fluorescent microscope. (**c**) absorption spectra of the green and red large area samples; (**d**) PL spectra of the green and red large area samples at exc of 350 nm.

Figure 8a,b show the large area fluorescence images of green light-emitted and red films realized with laser parameters suitable for green and red emissions (Table 5).

Their absorption and PL spectra are reported in Figure 8c,d. The absorption spectra of Figure 8c overlap with the spectrum of BZT that covers the features of the CdTe QDs, as already shown in Figure 3, and is present in both samples. The PL spectra (Figure 8d) suggests the presence of CdTe QDs, because the PL emissions bands are gaussian and relatively sharp (FWHM below 65 nm). 

These observations were repeated three times by using the same patterning conditions and the results, in terms of PL maximum and FWHM for green and red emissions, are summarized in Table 6.

The green emission is centered at 554 ± 12 nm (average ± 2 × average standard error for a confidence interval of 95%) with an FWHM of 58 ± 10 nm (average ± 2 × average standard error for a confidence interval of 95%). On the other hand, the red emission is centered at 629 ± 12 nm (average ± 2 × average standard error for a confidence interval of 95%) with an FWHM of 64 ± 2 nm (average ± 2 × average standard error for a confidence interval of 95%).

**Figure 9:**
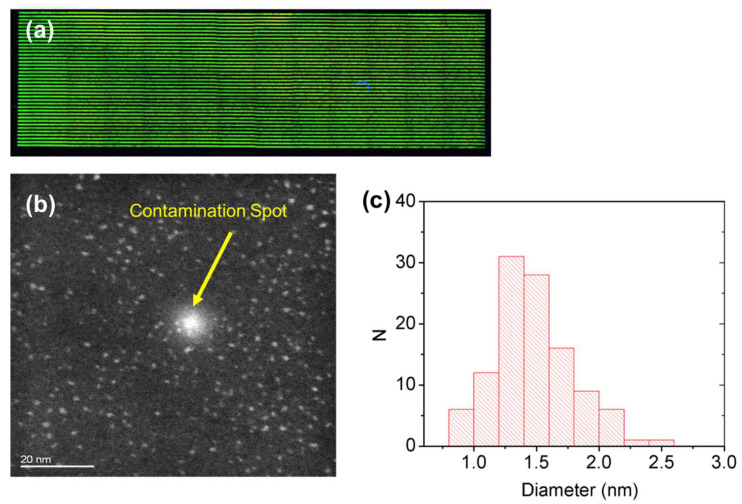
(**a**) Fluorescent microscope image of the large area samples obtained with the “green” laser parameters and the HAADF-STEM images (**b**) and the histogram of the particle size distribution (**c**). The image contrast is blurred by heavy contamination (white dot indicated by the yellow arrow), which is probably due to carbon formation under the electron beam.

**Figure 10:**
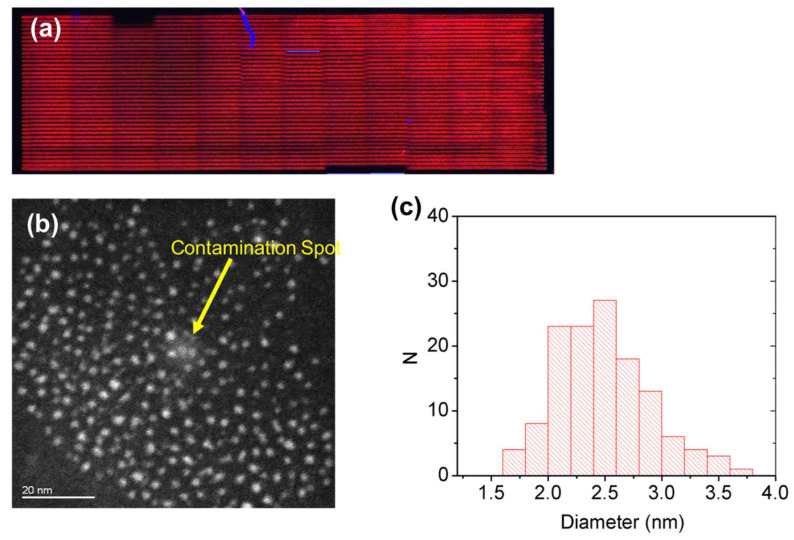
(**a**) Fluorescent microscope image of the large area samples obtained with the “red” laser parameters and (**b**) the HAADF-STEM image together with (**c**) the histogram of the particle size distribution. The image contrast is blurred by heavy contamination (white area indicated by the yellow arrow), which it is probably due to carbon formation under the electron beam.

For independent confirmation of the presence of CdTe QDs, the laser patterned samples were observed under a transmission electron microscope. The film was first treated with laser, by using the laser parameters shown in Table 5 (Figure 9 and Figure 10), then the film was dissolved in chloroform and observed with TEM.

The HAADF-STEM image of the green film (Figure 9) shows that the particles are crystalline (see Figure S6) within a size of 0.8 to 2.5 nm, and energy-dispersive spectroscopy (EDS) analysis confirmed the presence of Cd and Te (not shown). An estimation of the average diameter size and the size distribution of the QDs were obtained by the 130 QDs in different images that were measured.

The estimated average diameter is 1.4 nm with a standard deviation of 0.3 nm. 

Therefore, with a confidence interval of 99%, we assumed an average diameter value D_P_ = (1.4 ± 0.1) nm. As it is difficult to accurately reveal the edges of the particles, it is more conservative to assume an average diameter value of D_P_ = (1.4 ± 0.2).

Similarly, the presence of crystalline CdTe QDs was confirmed by TEM for the red sample (Figure 10). In particular, the particles are within the size range from 1.4 to 3.6 nm. The estimated average diameter is 2.4 nm with a standard deviation of 0.4 nm (measured on 130 QDs). Therefore, with a confidence interval of 99%, we assumed an average diameter value D_P_ = (2.4 ± 0.2) nm.

## 4. Discussion

It was already envisaged in the literature that formation of semiconductor QD size can be modulated in a polymer by laser irradiation; however, this effect was faint and for cadmium sulfide QDs a broad emission spectrum was observed [19,35]. A step forward is taken in this work, where the formation of different emission colors, from green to red, modulated by laser parameters, like average power and pulse frequency, is clearly shown. Few other papers show a direct laser patterning of CdTe QDs or CdSe@ZnS QDs. The laser source is used as a tool for structuring these already formed nanomaterials [36,37], but not for their synthesis, as in this work.

For direct laser patterning, the main concept of the work is to use precursors that, by thermal treatment, give rise to semiconductor CdTe QDs in solution and films [16]. However, to do this, the laser beam should induce a temperature rise in the matrix where the precursors are loaded. The energy uptake can take place if the QD precursors directly absorb the energy of the laser beam [17,23], or if the laser light is transformed into heat by adding a specific molecule which increases the absorption of the laser wavelength, as shown in Figure 3, by converting of the laser radiation in heat. In this work, this second option was followed by adding a UV sensitizer, i.e., BZT [31], because the CdTe QD precursors in the film did not sufficiently adsorb the laser radiation used, 355 nm.

The process of the laser patterning optimization shown in Figure 4 and Figure 5 demonstrates that by controlling some laser writing parameters, and keeping the film chemical formulation constant, it is possible to tune the QDs’ optical emission properties (sizes) from green to red.

These experiments open a series of interesting questions to which only preliminary answers can be given, based on current knowledge. Figure 4 describes how the precursors’ concentration affects PL emission: only when the Cd(ISA)_2_ concentration is 40 mg/mL (TOP-Te is fixed at 0.25 molar ratio) is it possible to obtain both green and red emissions by changing the laser parameters. At concentrations of Cd(ISA)_2_ greater than 40 mg/mL, there is a prevalence of red emission. suggesting that the higher concentration stimulates the growth of bigger QDs (red emitting ones) [38,39]. In this case, the variation of the concentration modulates the wavelength of the PL emission on the basis of the quantum size effect. On the contrary, in a similar work dealing with perovskite QDs’ formation by UV laser [40], the effect of increased concentration did not change the PL wavelength caused by the halide anion exchange, but did change photoluminescence intensity, due to the increased concentration of perovskite QDs.

Figure 5 shows that the role of the PMMA concentration in the QDs’ colors formation is just as important. At concentration of 100 mg/mL only green emission is observable, while at 300 mg/mL green and red emission lose their quality. For example, in the middle of the lines forming the green squares emission is destroyed, so the transferred energy is too much. Similarly, the lines of the “red” squares seem brown in the middle and yellow at the border. Only when the polymer concentration is 200 mg/mL are the conditions right for both red and green color formation. A possible explanation of the role of the PMMA in QDs’ color emission involves different properties of the polymer: (i) the precursors’ mobility within the polymer during QD formation [41], (ii) the thermal conductivity of PMMA, which can play a role affecting the growth of the QDs [42] (see below) and (iii) the polymer concentration that can influence QD optical properties modifying QD surface electronic states [43]. The first two properties modulate the growth of the QDs affecting the mobility of the atoms and the film temperature, while the third one is related to the interaction of the matrix with QD surface.

After this first set of experiments, two main working windows are identified for green and red-emitting QD formation: the green squares are obtained at doses of 30–130 J/cm^2^ at 20 kHz, while red ones are achieved with doses in the range 35–85 J/cm^2^ and pulse frequencies between 40–80 kHz. It is evident that the ranges of doses given to the films are quite similar and, in particular, the doses of the red range are included in intervals of doses of the green squares.

A further refinement of the conditions aiming to separate as effectively as possible the green patterns from the red ones, in terms of doses, was carried out by changing the number of loop counts (Figure 6 and Figure 7). The experiment shown in Figure 6 and Table 2, suggests that good green patterns, with well-defined laser lines, can be obtained only for high doses (loop counts 3, i.e., doses between 30–65 J/cm^2^ always at 20 kHz). Higher doses destroy the uniformity of the lines (see supporting information in Figure S2 and S3).

Figure 6 indicated also that at low doses (loop count 1 row A) the green is not obtained and, in some cases, a faint red emission is observed (Figure 6 squares A5–7). This evidence stimulated the authors to use the red square with just one loop count and high pulse frequencies to avoid any overlapping with the green pattern. Following this hypothesis, a matrix was carried out, by changing the power from 100% to 80% and pulse frequency from 100 kHz to 80 kHz, as shown in Table 3. Figure 7 confirms that in these conditions it was possible to obtain red emission from almost all the lines (from A to D range of doses between 7–13 J/cm^2^ with pulse frequency between 80 kHz and 100 kHz). However, in these conditions, the red patterns contain some green emissions also, as observed in Figure S4 (see supporting information). To overcome this difficulty a further refinement of the red matrix was carried out, modifying the speed of the beam from 100 mm/s to 10 mm/s, as shown in Figure S5 (supporting information). However, this change brings the dose of the red and green patterns in the same interval once again, as resumed in Table 5.

These numbers can give some suggestions to hypothesize a mechanism of CdTe QDs’ formation under laser treatment. The first consideration is that, in this type of experiment, the mechanism of QD formation is purely thermal, because without the sensitizer the film does not show any modification, and also because the role of the sensitizer is mainly that of absorbing light energy transforming it into thermal energy.

The second observation is that the strong difference in pulse frequency, correlated with the formation of red (80–100 kHz) and green (20 kHz) patterns while the laser doses are similar (Table 5), suggesting that pulse frequency has a crucial impact in film temperature during laser treatment. When the pulse frequency is low, namely 20 kHz, each laser pulse is separated from the next by about 50 μs, and this means that heating and cooling steps are sufficiently separated to achieve the correct temperature of the matrix, suitable to grow the green-emitting QDs.

On the other hand, at high frequencies (80–100 kHz), the temporal distance from the pulses is in the range 1–10 μs, and probably the temperature of the film is higher, because the sequence of the laser pulses is narrower, and the film has not enough time to cool down. 

**Figure 11:**
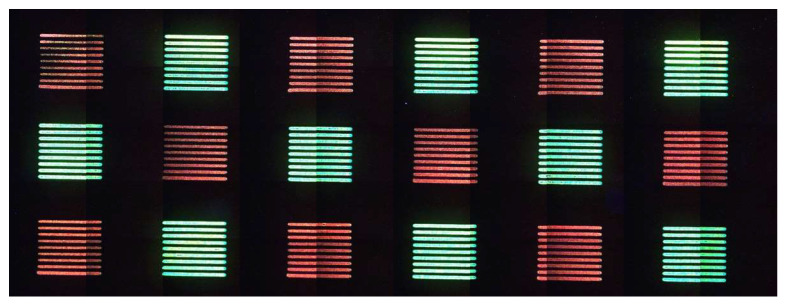
Fluorescence microscope image of the red/green chessboard obtained by using for red squares a laser power of 160 mW, pulse frequency of 100 kHz, speed 20 mm/s and loop count 1 that means a dose of 34.6 J/cm^2^. The green squares are obtained by using a laser power of 350 mW, pulse frequency 20 KHz, speed 100 mm/s and loop count 3 that means a dose of 44.8 J/cm^2^.

Even if the amount of the deposited energy is quite similar between the red and green patterns, its time distribution is crucial to get the right temperature for the QDs’ growth. For this reason, polymer thermal conductivity, depending on polymer type and concentration, also has an important impact on the correct color formation. Once identified, combining the green and red laser parameters with the chemical composition of the film makes it possible to obtain, within the same film, red and green areas, as reported in Figure 11.

The presence in the same film of green and red-emitting CdTe QDs is witness to the fact that keeping the chemical film composition constant, and just modulating the dose and repetition rate, makes it possible to obtain CdTe QDs emitting different colors. To our knowledge, this is the first result showing the laser synthesis of QDs’ emission from green to red in solid-state. Similar results are shown for perovskite QDs, with some differences from the present work. Indeed, Zhan et al. [40] obtained only red-emitting perovskite QDs in PMMA, while Dong [44] obtained only green-emitting perovskite QDs in the glass. The work of Xu et al. [45] showed the formation of perovskite nanocrystals from blue to green, but this emission was obtained after anion exchange in a solution of preformed perovskite QDs, so the film did not contain all the components to obtain different emission wavelengths.

A direct proof that PL emission is due to the presence of CdTe QDs derives from the study of PL emission spectra and transmission electron microscopy images of the patterned films. Two are evidence that CdTe PL emission is due to CdTe QDs: (i) PL emission ranges from green to red as was shown for similar CdTe QDs generated by heating in the polymer [16] and (ii) PL emission has a Gaussian shape that is relatively sharp; FWHM is 58 nm and 64 nm for green and red PL, respectively (Figure 8 and Table 6). This is typical of QDs with respect to organic substances that often have a broader photoluminescence spectrum. 

The TEM analysis confirms the presence of CdTe QDs with composition determined by the EDS and crystallinity by the Fast Fourier Transform (FFT) that indicates the planes (1,1,1) and (0,2,2) and their spatial distance (Figure S5 supporting information), with a relative size of green and red-emitting QDs of 1.4 ± 0.2 nm and 2.4 ± 0.2 nm, respectively (Figure 9 and Figure 10). The size of the nanoparticles is smaller for the green-emitting QDs with respect to the red ones as expected. However, the CdTe QDs obtained by pure thermal treatment in PMMA from the same precursors show a different size for green and red CdTe QDs and larger size distribution (2.0 ± 0.4 nm and 6.0 ± 0.9 nm, respectively) [16]. This means that the formation of the QDs by laser, even if stimulated by temperature rise, has a different mechanism of growth with respect to the pure thermal process. This phenomenon can be interpreted by considering that the reaction time with laser patterning is fractions of a second, while thermal growth takes several minutes (from 5 to 20 at 150 °C).

In addition, the fast rise of temperature can modify the shape and the surface of the QDs and, in particular, the polymer can be broken or modified and can bind at the QD surface, tuning the optical properties of the QDs like an organic ligand [43,46].

## 5. Conclusions

The main steps ahead described by this work, with respect to recent literature on the laser patterning of cadmium chalcogenide QDs, are (i) the clear demonstration that QDs can be generated by direct laser patterning and that (ii) their size, and hence their optical properties, can be modulated by tuning just the laser parameters, maintaining constant film chemical composition. The possibility to tune the CdTe spectrum from green to red through laser parameters is quite important and it being shown in such a clear way within a polymer is a new effect. Indeed, other authors with perovskite synthesized only green [44] or red [40] emitting nanocrystals or red or blue/green emitting nanocrystals by treating the sample in solution [45]. However, in this last case, it is necessary to add an external reagent (chloride anion replacing the pristine bromine anion), while in the present work all the chemicals are ready to form a multicolor emission.

Another step ahead concerns the mechanism of QDs’ formation. Indeed, only recently some aspects of the direct laser patterning of CdS QD precursors were clarified [25]. In the present work, the mechanism of QDs’ formation is purely thermal, because the experimental strategy has been built to have a pure thermic process formation of the CdTe QDs. Indeed, a UV sensitizer has been included in the film chemical formulation to convert the energy of light into thermal energy. Combining in a suitable way the laser parameters and the film chemistry it is possible to reach the proper temperature to grow green or red-emitting QDs. In this process, the laser pulse frequency (repetition rate) plays a central role in the modulation of film temperature, suggesting that the temporal distribution of energy in time is the key parameter stimulating red to green QD emission.

However, many aspects of the QDs’ growth through laser patterning have to be clarified: for example, (i) the size of the QDs and (ii) their intensity and stability.

The effects of the pulsed laser characteristics, as the size of the green and red-emitting QDs are grown by laser, is quite different from the size of the corresponding green and red-emitting QDs grown by pure thermal process [16,40].

Another important question is the CdTe QDs’ stability and intensity within the film. Indeed, the PL intensity is quite low (below 2%) and the film stability is rather poor (a few minutes under a blue light source). These two aspects are correlated, because CdTe QDs are not surrounded by a shell that stabilizes PL emission and increases its stability against oxygen and moisture. With respect to this issue, the selection of a suitable matrix (in terms of fixing surface defects) could help to solve these problems [47], as the presence of organic ligands [33] could help in stabilizing the formed QDs.

The result reached is quite important, because it demonstrates the possibility to tune QDs’ properties just by acting on UV laser parameters, and opens the possibility to apply laser patterning of nanomaterials as a complimentary technique of photolithography contact and ink-jet printing in several fields of applications. Moreover, this technique is used not only to prepare CdTe QDs, but also other nanomaterials like CdS [48] and CdSe [17], and recently perovskite [40,44] and graphene [49]. 

The use of an ever-increasing variety of materials means that the fields of application of nanomaterial laser patterning can expand in several directions: a possibility is to use this strategy to obtain color conversion filters for micro-displays and this work, indeed, stems from a project focused on micropatterning of color conversion filters (www.miledi-h2020.eu; accessed on 1 March 2018)) [50]. Other possible applications are shown with perovskite QDs to generate a micro-photodetection device [51] or sensors with graphene [49].

## 6. Patents

Some of the results of the work are included in the patent nr. 102020000020464 (26/8/2020).

## Figures and Tables

**Table 1:** Dose values as a function of the frequenccy of the laser pulse and percentage of the nominal laser power set in the instrument.

Dose (J/cm^2^)			Frequency ^a^ (kHz)							
			100	80	70	60	50	40	30	20
			1 ^c^	2	3	4	5	6	7	8
**Nominal Power ^b^ (%)**	**100**	**A ^d^**	28.7	39.7	47.4	57.1	69.2	84.5	103.6	127.5
	**80**	**B**	20.7	28.6	34.4	42.0	51.8	64.6	81.3	102.9
	**60**	**C**	16.3	18.4	20.5	23.7	28.8	36.8	49.4	69.0
	**40**	**D**	2.4	4.4	6.0	8.3	11.6	16.3	22.8	32.2
	**20**	**E**	1.0	1.0	1.1	1.3	1.6	2.2	3.3	5.6

^a^ frequency of the laser pulse; ^b^ percentage of the nominal laser power set in the instrument; ^c^ the numbers 1,2, …, 8 indicate the columns in Figure 4 and Figure 5; ^d^ the letters A, B, …, E indicate the lines in Figure 4 and Figure 5.

**Table 2:** Dose values as a function of the loop counts and the percentage of the nominal laser power set in the instrument.

Dose (J/cm^2^)										
**Nominal Power ^a^ (%)**			100	80	70	60	55	50	45	40
**Frequency ^b^ (kHz)**			20							
**Columns ^c^**			1	2	3	4	5	6	7	8
**Loop Count ^d^ (Rows**)	LC1	A	42.5	34.3	31.8	22.9	20.0	16.1	14.9	10.7
	LC2	B	85.0	68.6	63.7	45.8	40.0	32.3	29.9	21.5
	LC3	C	127.5	102.9	95.5	68.7	60.0	**48.4**	**44.8**	**32.2**
	LC4	D	170.1	137.3	127.4	91.6	80.0	**64.5**	**59.8**	**42.9**
	LC5	E	212.6	171.6	159.2	114.5	100.0	80.7	74.7	53.6

^a^ percentage of the nominal laser power set in the instrument; ^b^ frequency of the laser pulse; ^c^ the numbers 1,2, …, 8 indicate the columns in Figure 5; ^d^ the letters A, B, …, E indicate the lines in Figure 5.

**Table 3:** Summary of the laser parameters used to obtain red areas during direct laser writing process.

Dose (J/cm^2^)			Columns ^c^							
			1	2	3	4	5	6	7	8
**Rows ^d^**	**A**	**Dose**	9.6	9.7	9.9	10.0	10.2	10.3	10.5	10.7
		**Freq ^a^**	100	99	98	97	96	95	94	93
		**Power ^b^**	**100%**							
	**B**	**Dose**	10.8	11.0	11.2	11.4	11.6	11.8	12.0	12.2
		**Freq**	92	91	90	89	88	87	86	85
		**Power**	**100%**							
	**C**	**Dose**	12.4	12.6	12.8	13.0	9.2	9.1	8.9	8.8
		**Freq**	84	83	82	81	100	100	100	100
		**Power**	**100%**				**99%**	**98%**	**97%**	**96%**
	**D**	**Dose**	8.6	8.4	8.3	8.1	8.0	7.8	7.7	7.5
		**Freq**	**100 kHz**							
		**Power**	**95%**	**94%**	**93%**	**92%**	**91%**	**90%**	**89%**	**88%**
	**E**	**Dose**	7.4	7.2	7.1	7.0	6.8	6.7	6.5	6.4
		**Freq**	**100 kHz**							
		**Power**	**87%**	**86%**	**85%**	**84%**	**83%**	**82%**	**81%**	**80%**

^a^ frequency of the laser pulse; ^b^ percentage of the nominal laser power set in the instrument; ^c^ the numbers 1,2, …, 8 indicate the columns in Figure 7; ^d^ the letters A, B, …, E indicate the lines in Figure 7.

**Table 4:** Dose values as a function of the frequency of the speed parameters investigated.

**Power (%) ^a^**	**100**				
**Frequency (kHz) ^b^**	**100**				
**Loop Count**	**1**				
**Speed (mm/s)**	100	50	30	20	10
**Doses (J/cm^2^)**	9.6	19.1	31.9	47.8	95.6

^a^ percentage of the nominal laser power set in the instrument; ^b^ laser pulse frequency.

**Table 5:** Summary of the laser parameters used to obtain areas emitting selectively green or red during direct laser writing process.

	Power (mW)	Freq (kHz)	Speed (mm/s)	Loop Count	Dose (J/cm^2^)
Green	250–380	20	100	3	32.2–64.5
Red	150–225	100	20	1	31.9–47.8

**Table 6:** Position and FWHM values of the PL peaks as a function of the calculated dose values and the laser-writing parameters.

	PL Max (nm)	FWHM (nm)	Dose (J/cm^2^)	Freq (kHz)	Speed (mm/s)	LC
Green	554 ± 12	58 ± 10	32.2–48.4	20	100	3
Red	629 ± 12	64 ± 2	34.6–47.8	100	20	1

## Data Availability

Not applicable.

## Supplementary Materials

The following supporting information can be downloaded at: https://www.mdpi.com/article/10.3390/nano12091551/s1, Figure S1: control of direct laser patterning: film without BZT; Figure S2 details of the laser treatment on the formation of the green lines; Figure S3: Details of E8 and E1 squares of Figure S2; Figure S4: details of the effect of the laser patterning on the red lines at low doses; Figure S5: effect of the beam speed on red lines quality; Figure S6: HREM image of CdTe QDs.

## References

[1] Wiley Fundamental Principles of Optical Lithography: The Science of Microfabrication. https://www.wiley.com/en-us/Fundamental+Principles+of+Optical+Lithography%3A+The+Science+of+Microfabrication-p-9780470018934.

[2] Wolf M.P., Salieb-Beugelaar G.B., Hunziker P. (2018). PDMS with Designer Functionalities—Properties, Modifications Strategies, and Applications. Prog. Polym. Sci..

[3] Liu Y., Li F., Qiu L., Yang K., Li Q., Zheng X., Hu H., Guo T., Wu C., Kim T.W. (2019). Fluorescent Microarrays of in Situ Crystallized Perovskite Nanocomposites Fabricated for Patterned Applications by Using Inkjet Printing. ACS Nano.

[4] Wang Y., Fedin I., Zhang H., Talapin D.V. (2017). Direct Optical Lithography of Functional Inorganic Nanomaterials. Science.

[5] Park J.-S., Kyhm J., Kim H.H., Jeong S., Kang J., Lee S., Lee K.-T., Park K., Barange N., Han J. (2016). Alternative Patterning Process for Realization of Large-Area, Full-Color, Active Quantum Dot Display. Nano Lett..

[6] Lin Y., Zheng X., Shangguan Z., Chen G., Huang W., Guo W., Fan X., Yang X., Zhao Z., Wu T. (2021). All-Inorganic Encapsulation for Remarkably Stable Cesium Lead Halide Perovskite Nanocrystals: Toward Full-Color Display Applications. J. Mater. Chem. C.

[7] Sung S.H., Yoon H., Lim J., Char K. (2012). Reusable Stamps for Printing Sub-100 Nm Patterns of Functional Nanoparticles. Small.

[8] Antolini F., Orazi L. (2019). Quantum Dots Synthesis Through Direct Laser Patterning: A Review. Front. Chem..

[9] Stoian R., Colombier J.-P. (2020). Advances in Ultrafast Laser Structuring of Materials at the Nanoscale. Nanophotonics.

[10] Arnold C.B., Piqué A. (2007). Laser Direct-Write Processing. MRS Bull..

[11] Panfil Y.E., Oded M., Banin U. (2018). Colloidal Quantum Nanostructures: Emerging Materials for Display Applications. Angew. Chem. Int. Ed..

[12] Shirasaki Y., Supran G.J., Bawendi M.G., Bulović V. (2013). Emergence of Colloidal Quantum-Dot Light-Emitting Technologies. Nat. Photonics.

[13] Kim T.-H., Jun S., Cho K.-S., Choi B.L., Jang E. (2013). Bright and Stable Quantum Dots and Their Applications in Full-Color Displays. MRS Bull..

[14] Murray C.B., Norris D.J., Bawendi M.G. (1993). Synthesis and Characterization of Nearly Monodisperse CdE (E = Sulfur, Selenium, Tellurium) Semiconductor Nanocrystallites. J. Am. Chem. Soc..

[15] Todescato F., Fortunati I., Minotto A., Signorini R., Jasieniak J.J., Bozio R. (2016). Engineering of Semiconductor Nanocrystals for Light Emitting Applications. Materials.

[16] Carcione R., Limosani F., Antolini F. (2021). Cadmium Telluride Nanocomposite Films Formation from Thermal Decomposition of Cadmium Carboxylate Precursor and Their Photoluminescence Shift from Green to Red. Crystals.

[17] Limosani F., Carcione R., Antolini F. (2019). Formation of CdSe Quantum Dots from Single Source Precursor Obtained by Thermal and Laser Treatment. J. Vacuum Sci. Technol. B Nanotechnol. Microelectron. Mater. Process. Meas. Phenom..

[18] Antolini F., Burresi E., Stroea L., Morandi V., Ortolani L., Accorsi G., Blosi M. (2012). Time and Temperature Dependence of CdS Nanoparticles Grown in a Polystyrene Matrix. J. Nanomater..

[19] Resta V., Laera A.M., Camposeo A., Piscopiello E., Persano L., Pisignano D., Tapfer L. (2012). Spatially Confined CdS NCs in Situ Synthesis through Laser Irradiation of Suitable Unimolecular Precursor-Doped Polymer. J. Phys. Chem. C.

[20] Bansal A.K., Sajjad M.T., Antolini F., Stroea L., Gečys P., Raciukaitis G., André P., Hirzer A., Schmidt V., Ortolani L. (2015). In Situ Formation and Photo Patterning of Emissive Quantum Dots in Small Organic Molecules. Nanoscale.

[21] Smirnov A.A., Afanasiev A., Ermolaev N., Bityurin N. (2016). LED Induced Green Luminescence in Visually Transparent PMMA Films with CdS Precursor. Opt. Mater. Express.

[22] Agareva N., Smirnov A.A., Afanasiev A., Sologubov S., Markin A., Salomatina E., Smirnova L., Bityurin N. (2015). Properties of Cadmium-(Bis)Dodecylthiolate and Polymeric Composites Based on It. Materials.

[23] Fragouli D., Pompa P.P., Kalyva M., Caputo G., Tapfer L., Cingolani R., Athanassiou A. (2010). The Effect of Irradiation Wavelength on the Quality of CdS Nanocrystals Formed Directly into PMMA Matrix. J. Phys. Chem. C.

[24] Smirnov A.A., Afanasiev A., Gusev S., Tatarskiy D., Ermolaev N., Bityurin N. (2018). Exposure Dependence of the UV Initiated Optical Absorption Increase in Polymer Films with a Soluble CdS Precursor and Its Relation to the Photoinduced Nanoparticle Growth. Opt. Mater. Express.

[25] Bityurin N., Smirnov A.A. (2019). Model for UV Induced Growth of Semiconductor Nanoparticles in Polymer Films. Appl. Surf. Sci..

[26] Jasieniak J., Califano M., Watkins S.E. (2011). Size-Dependent Valence and Conduction Band-Edge Energies of Semiconductor Nanocrystals. ACS Nano.

[27] Haram S.K., Kshirsagar A., Gujarathi Y.D., Ingole P.P., Nene O.A., Markad G.B., Nanavati S.P. (2011). Quantum Confinement in CdTe Quantum Dots: Investigation through Cyclic Voltammetry Supported by Density Functional Theory (DFT). J. Phys. Chem. C.

[28] Mulko L., Soldera M., Lasagni A.F. (2022). Structuring and Functionalization of Non-Metallic Materials Using Direct Laser Interference Patterning: A Review. Nanophotonics.

[29] Broglia M.F., Suarez S., Soldera F., Mücklich F., Barbero C.A., Bellingeri R., Alustiza F., Acevedo D. (2014). Direct Laser Interference Patterning of Polystyrene Films Doped with Azo Dyes, Using 355 nm Laser Light. Appl. Surf. Sci..

[30] Dorfs D., Franzl T., Osovsky R., Brumer M., Lifshitz E., Klar T.A., Eychmüller A. (2008). Type-I and Type-II Nanoscale Heterostructures Based on CdTe Nanocrystals: A Comparative Study. Small.

[31] Lafleur S.S.D., Shen L., Kamphuis E.J.T.W., Houben S.J.A., Balzano L., Severn J.R., Schenning A.P.H.J., Bastiaansen C.W.M. (2019). Optical Patterns on Drawn Polyethylene by Direct Laser Writing. Macromol. Rapid Commun..

[32] García-Rodríguez R., Liu H. (2013). Solution Structure of Cadmium Carboxylate and Its Implications for the Synthesis of Cadmium Chalcogenide Nanocrystals. Chem. Commun..

[33] Kirkwood N., Monchen J.O.V., Crisp R.W., Grimaldi G., Bergstein H.A.C., du Fossé I., van der Stam W., Infante I., Houtepen A.J. (2018). Finding and Fixing Traps in II–VI and III–V Colloidal Quantum Dots: The Importance of Z-Type Ligand Passivation. J. Am. Chem. Soc..

[34] Antolini F., Ortolani L. (2017). CdTe Quantim Dots Nanocomposite Films Obtained by Thermal Decomposition of Precursors Embedded in Polymeric Matrix. Physics, Chemistry and Application of Nanostructures.

[35] Fragouli D., Laera A.M., Pompa P.P., Caputo G., Resta V., Allione M., Tapfer L., Cingolani R., Athanassiou A. (2009). Localized Formation and Size Tuning of CdS Nanocrystals upon Irradiation of Metal Precursors Embedded in Polymer Matrices. Microelectron. Eng..

[36] Xu B.-B., Zhang Y.-L., Zhang R., Wang L., Xiao X.-Z., Xia H., Chen Q.-D., Sun H.-B. (2013). Programmable Assembly of CdTe Quantum Dots into Microstructures by Femtosecond Laser Direct Writing. J. Mater. Chem. C.

[37] Ritacco T., Lio G.E., Xu X., Broussier A., Issa A., Giocondo M., Bachelot R., Blaize S., Couteau C., Jradi S. (2021). Three-Dimensional Photoluminescent Crypto-Images Doped with (CdSe)ZnS Quantum Dots by One-Photon and Two-Photon Polymerization. ACS Appl. Nano Mater..

[38] Vreeland E.C., Watt J., Schober G.B., Hance B.G., Austin M.J., Price A.D., Fellows B.D., Monson T.C., Hudak N.S., Maldonado-Camargo L. (2015). Enhanced Nanoparticle Size Control by Extending LaMer’s Mechanism. Chem. Mater..

[39] Wang F., Richards V.N., Shields S.P., Buhro W.E. (2014). Kinetics and Mechanisms of Aggregative Nanocrystal Growth. Chem. Mater..

[40] Zhan W., Meng L., Shao C., Wu X., Shi K., Zhong H. (2021). In Situ Patterning Perovskite Quantum Dots by Direct Laser Writing Fabrication. ACS Photonics.

[41] Liu Y., Zhang X. (2021). Mobility of Small Molecules in Solid Polymer Film for π-Stacked Crystallization. Crystals.

[42] Lu Y., Liu J., Xie X., Cahill D.G. (2016). Thermal Conductivity in the Radial Direction of Deformed Polymer Fibers. ACS Macro Lett..

[43] Karan S., Majumder M., Mallik B. (2012). Controlled Surface Trap State Photoluminescence from CdS QDs Impregnated in Poly(Methyl Methacrylate). Photochem. Photobiol. Sci..

[44] Huang X., Guo Q., Yang D., Xiao X., Liu X., Xia Z., Fan F., Qiu J., Dong G. (2020). Reversible 3D Laser Printing of Perovskite Quantum Dots inside a Transparent Medium. Nat. Photonics.

[45] Xu X., Dong Y., Zhang Y., Han Z., Liu J., Yu D., Wei Y., Zou Y., Huang B., Chen J. (2022). High-Definition Colorful Perovskite Narrowband Photodetector Array Enabled by Laser-Direct-Writing. Nano Res..

[46] Kahmann S., Loi M.A. (2020). Trap States in Lead Chalcogenide Colloidal Quantum Dots—Origin, Impact, and Remedies. Appl. Phys. Rev..

[47] Prudnikau A., Shiman D.I., Ksendzov E., Harwell J., Bolotina E.A., Nikishau P.A., Kostjuk S.V., Samuel I.D.W., Lesnyak V. (2021). Design of Cross-Linked Polyisobutylene Matrix for Efficient Encapsulation of Quantum Dots. Nanoscale Adv..

[48] Lipatiev A.S., Shakhgildyan G.Y., Vetchinnikov M.P., Lee H., Heo J., Lotarev S.V., Sigaev V.N. (2022). Direct Precipitation of CdS Nanocrystals in Glass by Ultrafast Laser Pulses. Mater. Lett..

[49] Vivaldi F.M., Dallinger A., Bonini A., Poma N., Sembranti L., Biagini D., Salvo P., Greco F., Di Francesco F. (2021). Three-Dimensional (3D) Laser-Induced Graphene: Structure, Properties, and Application to Chemical Sensing. ACS Appl. Mater. Interfaces.

[50] Sajjad M.T., Bansal A.K., Antolini F., Preis E., Stroea L., Toffanin S., Muccini M., Ortolani L., Migliori A., Allard S. (2021). Development of Quantum Dot (QD) Based Color Converters for Multicolor Display. Nanomaterials.

[51] Chou S.S., Swartzentruber B.S., Janish M.T., Meyer K.C., Biedermann L.B., Okur S., Burckel D.B., Carter C.B., Kaehr B. (2016). Laser Direct Write Synthesis of Lead Halide Perovskites. J. Phys. Chem. Lett..

