# Supplementary material for: Direct Laser Patterning of CdTe QDs and Their Optical Properties Control through Laser Parameters
Source: Nanomaterials (Basel). 2022 May 4;12(9):1551. doi: 10.3390/nano12091551 (PMC9103134; doi:10.3390/nano12091551)
Supplement: Supplementary file 1 [file nanomaterials-12-01551-s001.zip › nanomaterials-1680860 supplementary new.pdf]

nanomaterials

MDPI

Supplementary Materials

Direct Laser Patterning of CdTe QDs and Their Optical Properties Control through Laser Parameters

Francesco Antolini [1,*], Francesca Limosani [2,3] and Rocco Carcione [4]

[1] Fusion and Technologies for Nuclear Safety and Security Department, Physical Technologies for Safety and Health Division, Photonics Micro and Nanostructures Laboratory, ENEA C.R. Frascati, via Enrico Fermi 45, 00044 Frascati (RM), Italy

[2] Department of Information Engineering, Polytechnic University of Marche, Via Brecce Bianche, 1, 60131 Ancona, Italy; f.limosani@univpm.it

[3] INFN-National Laboratories of Frascati, Via Enrico Fermi, 54, 00044 Frascati, Italy;

[4] Consiglio Nazionale delle Ricerche, Institute of Materials for Electronics and Magnetism (CNR-IMEM), Parco Area delle Scienze 37A, 43124 Parma, Italy; rocco.carcione@imem.cnr.it

* Correspondence: francesco.antolini@enea.it

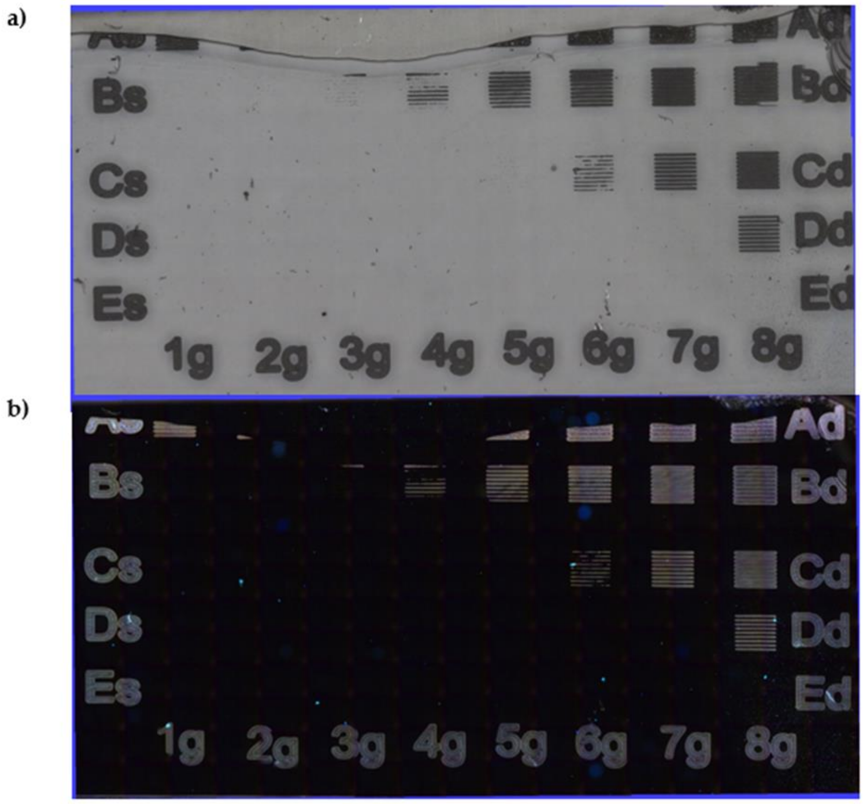

Figure S1. Control of direct laser patterning: film without BZT. (**a**) The image shows the observed results of the microscope under normal light of a sample treated with laser where cadmium and tellurium precursors are absent. (**b**) The image shows the same film under UV excitation. The squares do not emit any color, while the "white" emission of the squares observed is due to environmental light during the microscopic observation.

This type of experiment is aimed at checking if the color formation is due to any material treatment not involving Cd, Te.

The film observed in figure S1 was prepared depositing a solution of $Cd(ISA)_2$ 0.059 mmol/ml, TOP-Te 0.0147 mmol/ml, PMMA 100 mg/ml and BZT 2 mg/ml, in chloroform by spin coating.

The spin coating conditions were 1000 rpm for 45 sec and the volume deposited was 100 μl.

The laser patterning experiment was carried out by using the general matrix with 1 loop count at 100 mm/sec beam speed.

Another control experiment was carried out removing the BZT from the solution. In this condition the film was not structured at all by the laser (not shown) because it was transparent to UV laser radiation.

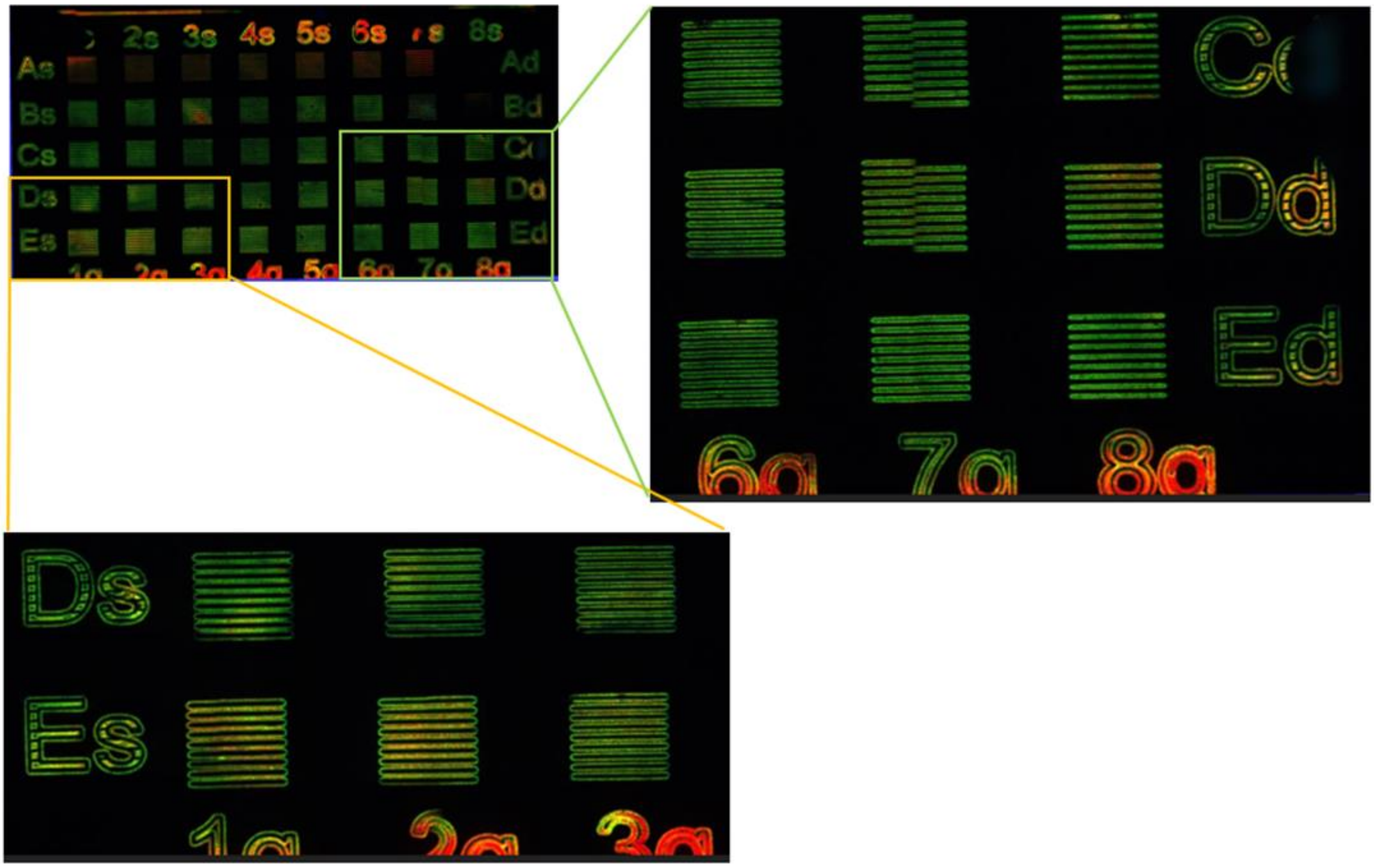

Figure S2. Details of the laser treatment on green line formation.

The squares D1-3 ed E1-3 are formed by lines that are not emissive in their central part, indicating that the PL was destroyed (see Figure S3 E1 square enlarged). The squares D6-8 and E6-8 were formed by lines that are homogeneous, in particular the ones in column 8 (see Figure S3 E8 square enlarged).

The laser patterning experiment was carried out by using the general matrix, built as in table 2.

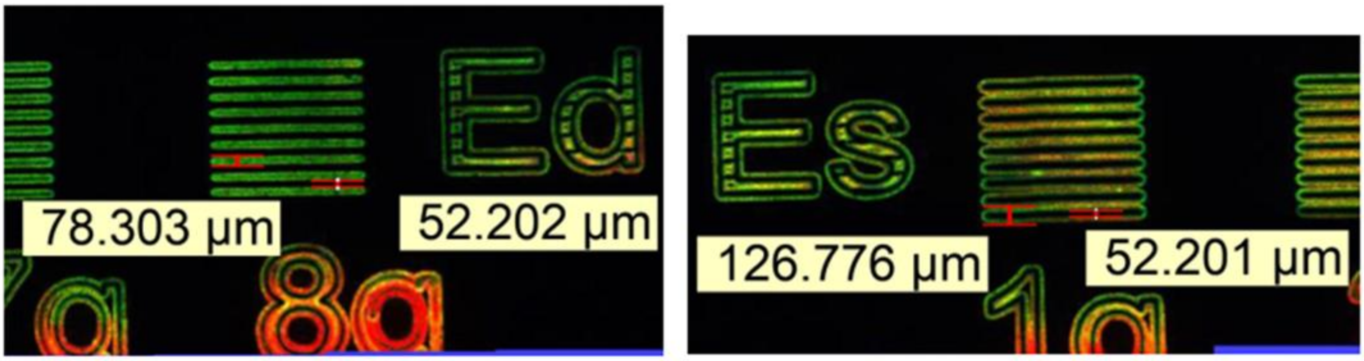

Figure S3. Details of the E8 and E1 squares of Figure S2.

Magnified image of the square E1 below of figure S2 (right) with the size of line of about 125 μm and central dark area of 52 μm. No distance between the lines was observed.

Magnified image of square E8 (left), where the line thickness is about 80 μm and the distance between the lines about 50 μm.

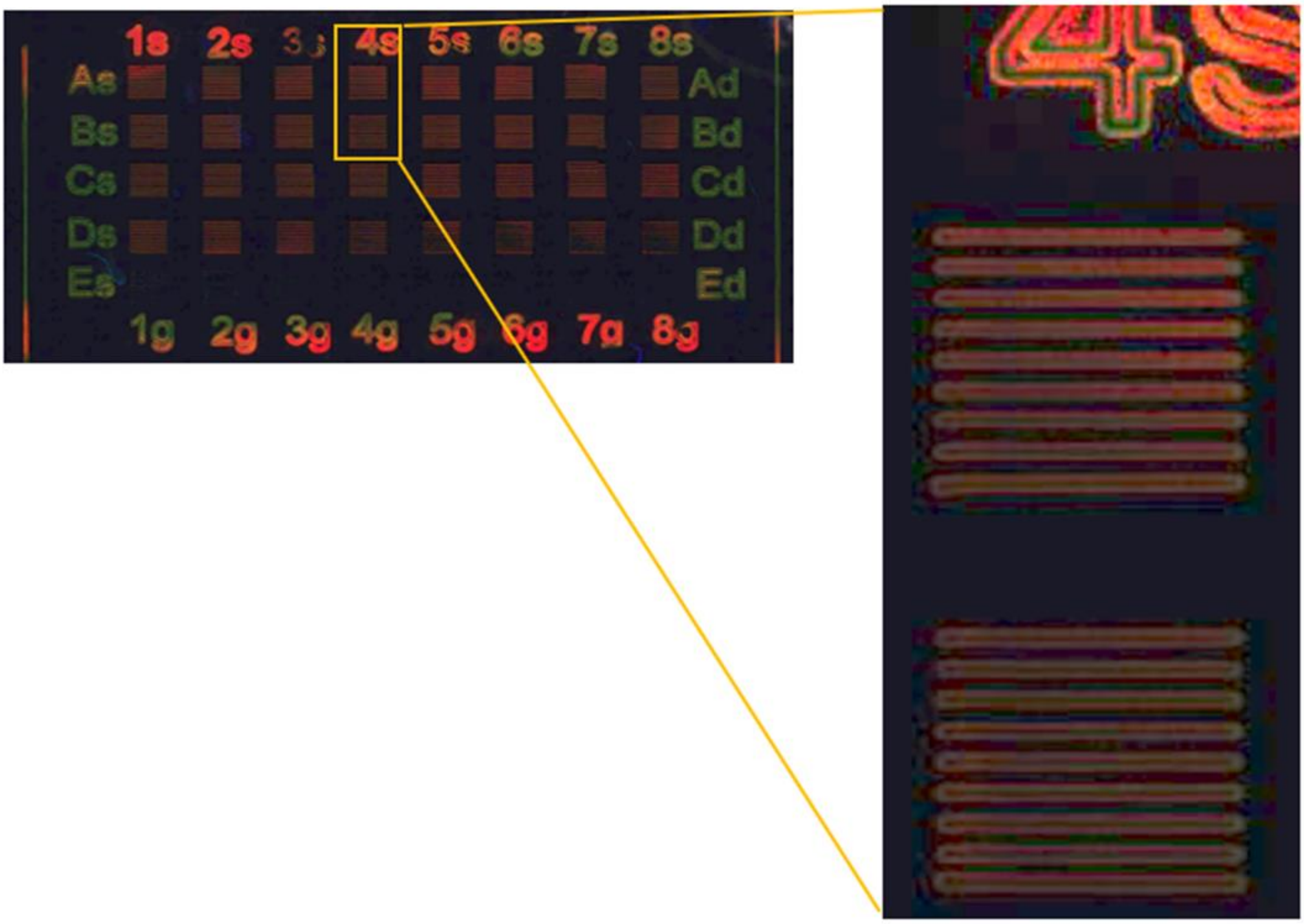

Figure S4. Details of the effect of the laser patterning on the red lines at low doses. On the left (A) is the matrix of the laser treatment and is the same as figure 6 in the text. The image on the right (B) shows the squares included in the yellow box that are enlarged to show the effect of the laser: the color of the lines is not pure red as there are still green areas around the red.

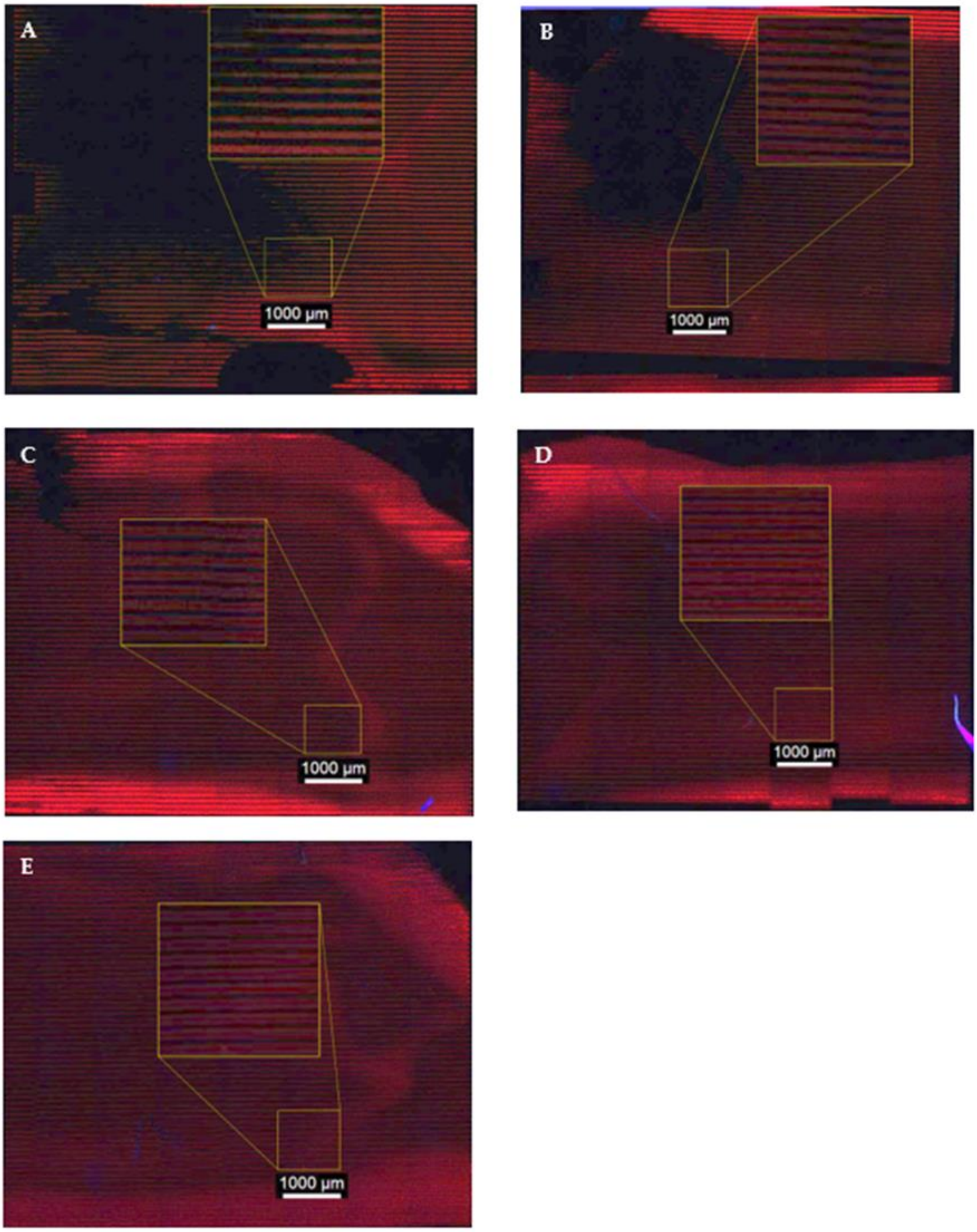

Figure S5. Effect of the beam speed on red lines quality. The figure shows the different effects of laser speed during the red writing on large area samples: (**A**) speed 100 mm/sec, (**B**) 50 mm/sec, (**C**) 30 mm/sec, (**D**) 20 mm/sec, (**E**) 10 mm/sec.

The optical analysis shows that at a speed of 100 mm/s and 50 mm/s there was still some green presence and below 30 mm/sec this presence was lower.

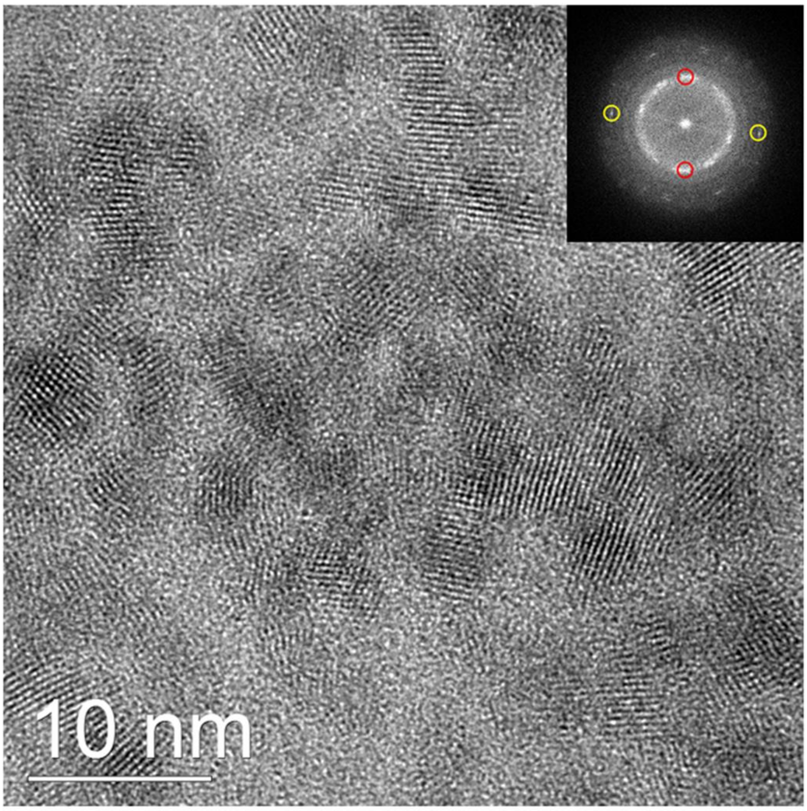

Figure S6. HREM image of CdTe QDs. HREM image of the green-emitting CdTe QDs generated upon laser treatment. HREM image of the CdTe nanoparticles. In the inset, the Fast Fourier Transform (FFT) of the same image is shown. The red circles indicates spatial periodicity of 0.374 nm, corresponding to (1,1,1) planes of the CdTe, while the yellow ones are related to (0,2,2) planes (d = 0.229 nm).
